# Characterization of Physicochemical Properties, Bioactivities, and Sensory Attributes of Sea Buckthorn–Fava Bean Composite Instant Powder: Spray-Drying Versus Freeze-Drying Coupled with Carriers

**DOI:** 10.3390/foods13233944

**Published:** 2024-12-06

**Authors:** Shi Li, Xizhe Fu, Jing Wen, Lin Jiang, Liheng Shao, Yinglin Du, Chunhui Shan

**Affiliations:** 1 Engineering Research Center of Storage and Processing of Xinjiang Characteristic Fruits and Vegetables, Ministry of Education, School of Food Science, Shihezi University, Shihezi 832000, China; 18990942689@163.com (S.L.); xizhefu@zju.edu.cn (X.F.); 15072468279@163.com (J.W.); 18862187580@163.com (L.J.); 13384789560@163.com (L.S.); 2 Key Laboratory of Processing and Quality and Safety Control of Specialty Agricultural Products (Co-Construction by Ministry and Province), Ministry of Agriculture and Rural Affairs, School of Food Science, Shihezi University, Shihezi 832000, China; 3 Key Laboratory for Food Nutrition and Safety Control of Xinjiang Production and Construction Corps, School of Food Science, Shihezi University, Shihezi 832000, China

**Keywords:** spray-drying, freeze-drying, maltodextrin, inulin, sea buckthorn, fava bean, beverage

## Abstract

Foods and beverages with health benefits have become increasingly popular with consumers, and fruits and legumes are considered good sources of nutrients. In this study, sea buckthorn and fava bean were used as the main raw materials to prepare sea buckthorn–fava bean composite instant powder (S-FCP). Different drying methods (spray-drying (SD) and freeze-drying (FD)) combined with carriers (maltodextrin (MD) and inulin (INU)) were involved to investigate their effects on physicochemical properties, functional properties, and sensory attributes of instant powder. The results showed that FD better protected the color of the S-FCP and produced particles possessing more porous structures compared to SD; FD-INU (freeze-dried-inulin) had the shortest dissolution time and the largest solubility. In addition, FD-INU had the highest total phenolic and total flavonoid contents and the strongest antioxidant capacity, and FD-INU had better overall organoleptic properties and hypoglycemic potential. Therefore, FD and the use of INU as a carrier are more suitable for the production of the S-FCP. This work provides a promising approach for developing a high-valued instant powder beverage composed of sea-buckthorn/broad bean, which also contributes to the development of the functional food industry.

## 1. Introduction

Sea buckthorn (*Hippophae rhamnoides* L.) is a deciduous shrub mainly found in the cold and arid regions of Asia, Europe, and North America, and is a medicinal and edible homologous plant. Sea buckthorn berries are rich in vitamins, essential fatty acids, organic acids, polysaccharides, phenolics, steroids, triterpenoids, and other biologically active substances, with antioxidant, hypoglycemic, anti-inflammatory, cardioprotective, and anti-atherosclerotic activities, which have been commonly used in the development of health care foods and dietary nutritional supplements [1,2]. Fava bean (*Vicia faba* L.) is a cool-season annual legume that grows in a variety of climatic conditions. As a high-protein, starch-rich, low-fat, and dietary fiber-rich legume, its glycemic index is lower than that of most grains, and it has hypoglycemic and hypolipidemic effects, making it an ideal low-energy meal replacement ingredient [3,4,5]. However, the sour taste of sea buckthorn and the unpleasant smell of fava beans limit the application of their related products, which demand formulation compounding to improve the flavor.

In recent years, the correlation between diet and health has drawn increasing attention, and traditional dietary patterns have gradually changed towards food with clean labels and functionality [6]. Among functional foods, functional beverages have the fastest value addition in the global market and are expected to grow to USD 208.13 billion by 2024, which promotes the research and development of related products [7]. Among functional beverages, solid beverages are one of the most popular forms of products, possessing considerable advantages including relatively low transportation cost and storage expenses and extended shelf life. Spray drying (SD) and freeze-drying (FD) are the most widely used drying technologies [8]. SD is a thermal drying technology, widely used in the food industry due to its low cost, high efficiency, and mature process [9]. Unlike SD, FD is a low-temperature drying technology which is characterized by low temperature and low oxygen and can effectively protect the nutrients and color of the products [10]. However, pure fruit juice powder is more hygroscopic and prone to agglomeration and adhesion to the surface of the dryer due to the low glass transition temperature of low-molecular-weight sugars and acids in the matrix [8,11]. Therefore, natural food ingredients rich in high-molecular-weight substances, such as maltodextrin (MD) and inulin (INU), are usually involved to reduce the hygroscopicity and improve the thermal stability of products. To address the concerns of nutrient supplementation and organoleptic characteristics, a small amount of millet was also incorporated for instant powder processing.

Many studies have shown that the carrier and drying method can significantly affect the physicochemical properties and biological activity of powders, e.g., sea buckthorn juice powder with added inulin has greater water retention than the same powder with added maltodextrin [8]; Compared with SD mango powder, the rheological behavior of the redissolved pulp of FD mango powder is more similar to that of the original mango pulp [12]. There have been a small number of studies on juice–legume composite beverages. Potter et al. [13] compounded blueberries and soybeans to make a beverage that masked the beany flavor and also had a strong antioxidant capacity; Morales-De la Peña et al. [14] developed a red prickly pear–soymilk composite beverage; and Rodríguez-Roque et al. [15] compounded composite juice with soybean beverage and found that the juice–soymilk beverage showed higher bioaccessibility of its hydrophilic ingredients than its lipophilic ones. However, no studies have been conducted on the effect of drying methods and carriers on juice–legume composite instant powder. Therefore, this study aimed to investigate the effects of different drying methods (spray drying and freeze drying) and carriers (maltodextrin and inulin) on the physicochemical properties (color, particle size, microstructure, contact angle, wettability, solubility, moisture content, water distribution, and functional group characteristics), basic composition (total phenolic content and total flavonoid content), antioxidant capacity (DPPH and ABTS radical scavenging capacity), and sensory attributes, as well as the phenolic content, of the final product and the hypoglycaemic effect of the S-FCP, to provide new insight and a theoretical basis for the development of the functional instant powder industry.

## 2. Materials and Methods

### 2.1. Raw Materials and Reagents

Mature sea buckthorn berries (variety: ShenQiuHong; average fruit diameter: 8.66 ± 0.57 mm; individual fruit weight: 0.28 ± 0.11 g), dried fava beans (Individual grain length: 22.38 ± 0.64 mm; Single grain weight: 1.70 ± 0.22 g; moisture content: 10.92 ± 0.38% (m/m)), and millet (moisture content: 8.88 ± 0.25% (m/m)) were purchased from the local market in Shihezi, Xinjiang, and stored in a −18 °C refrigerator. Maltodextrin (DE = 16–20) and inulin (food grade) were purchased from Henan Wanbang Chemical Science and Technology Co., Ltd. (Henan, China). Other analytical grade chemicals, including sodium hydroxide, sodium carbonate, anhydrous ethanol, 1,1-diphenyl-2-picrylhydrazyl (DPPH), 2,2′-azino-bis-3-ethylbenzothiazoline-6-sulfonic acid (ABTS), α-glucosidase, and α-amylase, etc., and the chromatography grade chemicals, including gallic acid, protocatechuic acid, catechin, and methanol, etc., were purchased from Shanghai Maclin Biochemical Technology Co., Ltd. (Shanghai, China). Ultra-pure water was used throughout the experiment.

### 2.2. Preparation of Fresh Sea Buckthorn–Fava Bean Composite Beverage

The sea buckthorn berries were thawed, and the broken and moldy berries were picked out before the remaining berries were washed with water. The washed sea buckthorn berries were crushed and pulped and then filtered through 4 layers of gauze (pore size: 1 mm × 1 mm) to obtain sea buckthorn juice. Dried fava beans were soaked in water for 16 h at a ratio of 1:3 (dried fava beans:water, *w*/*w*) and then peeled. The peeled fava beans, millet, and water were pulped with a soy milk machine (MJ-PB8G2-071, Midea Group Co., Ltd., Foshan, Guangdong, China) at the ratio of 1:0.12:9 (peeled fava beans:millet:water, *w*/*w*/*w*) to obtain fava bean pulp. Sea buckthorn juice was mixed with fava bean pulp at the ratio of 1:2 (sea buckthorn juice:fava bean pulp, *w*/*w*) and homogenized with a high-pressure homogenizer (SY-100, Shanghai Shunyi Experimental Equipment Co., Ltd., Shanghai, China) for 5 min (20 MPa) and the sea buckthorn–fava bean composite beverage was obtained. This preparation process was obtained by using bean-to-water ratio, millet addition, and sea buckthorn juice addition as single factors, and the response surface optimization test was carried out based on a fuzzy mathematical sensory score as a response value.

### 2.3. Preparation of Sea Buckthorn–Fava Bean-Carriers Complex

The prepared sea buckthorn–fava bean composite beverage was divided into 3 portions, 1 portion without carrier as a blank group (CK), and the remaining 2 portions with 20% (*w*/*w*) maltodextrin (MD) and 5% (*w*/*w*) inulin (INU). The addition amount was based on a preliminary single-factor test with the powder collection rate, water content, and solubility of the S-FCP as response values.

### 2.4. Drying of Sea Buckthorn–Fava Bean Composite Instant Powder

The CK, MD, and INU groups were further divided into 2 portions, respectively. One portion was spray-dried (SD) and the other portion was freeze-dried (FD) to prepare the S-FCP.

Spray drying (SD) was carried out using a laboratory spray dryer (QFN-6000Y, Ningbo Xinyi Ultrasonic Equipment Co., Ltd., Ningbo, China) with an inlet temperature of 180 °C and a feed flow rate of 40 mL/min. Before freeze-drying (FD), the sea buckthorn–fava bean composite beverage was spread flat in a glass dish to control the thickness of the sample at 10 mm, and then the samples were pre-frozen at −80 °C for 12 h. The freeze-drying was performed in a freeze-dryer (SCIENTZ-20F/B, Ningbo SCIENTZ Biotechnology Co., Ltd., Ningbo, China) for 36 h with a vacuum of 0.01 mbar and a cold trap temperature of −55 °C. The freeze-dried samples were placed in a small pulverizer (SMF01, Zhejiang Supor Co., Ltd., Hangzhou, China) with a power of 500 W and ground for 30 s to obtain FD powder samples. All the spray-dried and freeze-dried samples were divided into three portions, vacuum-sealed, and stored at −20 °C for further analysis. SD and FD samples were collected, encapsulated in vacuum-sealed bags, and stored at −20 °C.

### 2.5. Determination of Physicochemical Properties of S-FCP

#### 2.5.1. Color Parameters

A colorimeter (WSC-2S, Shanghai Yidian Physical and Optical Instrument Co., Ltd., Shanghai, China) was used to determine the brightness (L*), red value (a*), and yellow value (b*). First, the black zero cup and working whiteboard were used for zero adjustment and calibration. After calibration, 1 mL of fresh sea buckthorn–fava bean composite beverage was measured in the sample cup. Thereafter, 1 g of the S-FCP was spread evenly in the solid powder measuring dish, and the top lid of the dish with cylindrical protrusions was slowly tightened to obtain a solid sheet of uniform thickness for measurement. The total color difference was calculated as follows:

(1) $$∆E=\sqrt{{\left(L_{o}^{*}-L^{*}\right)}^{2}+{\left(a_{o}^{*}-a^{*}\right)}^{2}+{\left(b_{o}^{*}-b^{*}\right)}^{2}}$$

where $L_{o}^{*}$, $a_{o}^{*}$, and $b_{o}^{*}$ are the colorimetric values of the fresh sea buckthorn–fava bean composite beverage and $L^{*}$, $a^{*}$, and $b^{*}$ are the colorimetric values of the S-FCP.

#### 2.5.2. Particle Size

The particle size distribution of the S-FCP dispersed in water was measured using a laser particle size analyzer (winner2000ZD, Jinan Micro-Nano Particle Instrument Co., Ltd., Jinan, China). The average volume diameter, usually expressed as D_[4,3]_, was calculated according to Equation (2).

(2) $$D_{\left[4,3\right]}=\frac{\sum_{i=1}^{n}n_{i}d_{i}^{4}}{\sum_{i=1}^{n}n_{i}d_{i}^{3}}$$

where *n_i_* denotes the number of particles of diameter *d_i_*.

The spanning index is used to characterize the particle size distribution; the closer the value of the span is to 1, the narrower the particle size distribution. The span index is calculated as follows:

(3) $$span=\frac{D_{90}-D_{10}}{D_{50}}$$

where *D*_90_, *D*_50_, and *D*_10_ denote the particle size corresponding to the cumulative particle size percentages of 90%, 50%, and 10%, respectively.

#### 2.5.3. Scanning Electron Microscope (SEM)

The microstructure of the S-FCP was observed using a scanning electron microscope (SU8010, Hitachi Co., Ltd., Tokyo, Japan) at an accelerating voltage of 5 kV and magnifications of 200× and 2000×.

#### 2.5.4. Contact Angle

The indirect contact angle between the powder and deionized water was measured by the seated drop method using a contact angle interfacial viscoelastic measuring instrument (VCA25, Beijing Audrino Instrument Co., Ltd., Beijing, China). A tablet press was used to press the sample into a thin slice on the carrier table, and a syringe containing deionized water was placed on the launching stand to record the whole process of contact between the droplet and the sample, and the contact angle of the sample was measured.

#### 2.5.5. Wetting Time

The wetting time of the S-FCP was determined according to the method reported by Tahiya Qadri et al. [16]. In brief, a glass funnel with an outer tube diameter of 1 cm was fixed above the beaker containing 100 mL of deionized water at 25 °C, then 1 g of powder was added to the beaker through the funnel, and the time required for the powder to be completely wetted was recorded with the help of a stopwatch.

#### 2.5.6. Solubility

The solubility of S-FCP was determined as previously described with slight modification [17]. A total of 1 g of the powder sample was dissolved in 100 mL of deionized water with 5 min stirring at 500 rpm for complete dissolution. The solution was centrifuged at 3000× *g* rpm for 5 min, and the supernatant (25 mL) was dried at 105 °C in a drying oven (BPG-9240A, Shanghai Yihang Scientific Instrument Co., Ltd., Shanghai, China) until constant weight, and the solubility was calculated as follows:

(4) $$Solubility(\%)=\frac{weight\;of\;dried\;supernatant}{weight\;of\;the\;sample}\times 100$$

#### 2.5.7. Moisture Content

Based on the method adopted by Siccama et al. [18], the mass loss was determined during drying at 105 °C until the sample mass was constant. The formula for calculating the moisture content of the sample is as follows:

(5) $$Moisture\;content\left(\%\right)=\frac{M_{1}-M_{2}}{M_{1}}\times 100$$

where *M*_1_ is the mass of the sample before drying (g) and *M*_2_ is the mass of the sample after drying (g).

#### 2.5.8. Low Field Nuclear Magnetic Resonance (LF-NMR)

The transverse relaxation time (T_2_) of the S-FCP was determined using an LF-NMR analyzer (NMI20-015V-I, Suzhou Newmax Analytical Instruments Co., Ltd., Suzhou, China). The sample was put into a glass sample tube, and measurement was carried out to capture the decay signal using the CPMG (Carr Purcell-Meiboom-Gill) sequence. The CPMG parameters were set as follows: 90° pulse width of 12.8 μs, 180° pulse width of 22.4 μs, waiting time of 700 ms, echo time of 0.2 ms, and echo number of 5000. The scans were repeated 8 times to finally construct the T_2_ distribution curve.

#### 2.5.9. Fourier Transform Infrared Spectroscopy (FT-IR)

The S-FCP was ground with KBr (1%, *w*/*w*) pressed into tablets, and then analyzed using a Fourier transform infrared spectrometer (Bruker Vertex 70 V, Shanghai Erdie Instrument Science and Technology Co., Ltd., Shanghai, China). The spectral band range was 4000–400 cm^−1^ with a resolution of 4 cm^−1^.

### 2.6. Determination of Bioactive Components, Antioxidant Capacity, and Hypoglycaemic Effect of S-FCP

#### 2.6.1. Total Phenolic Content (TPC)

TPC was determined based on the method previously reported [19]. Briefly, 1 g of sample powder was mixed with 25 mL of 80% methanol solution, shaken at room temperature for 30 min, centrifuged at 6000× *g* rpm for 15 min, and the supernatant was collected and stored at −20 °C. A total of 1 mL of extract was mixed with 5 mL of Folin–Ciocalteu reagent (10%, *v*/*v*) and 4 mL of 7.5% Na_2_CO_3_ solution. The mixture was kept at room temperature for 1 h, and the absorbance was measured at 765 nm. The results were expressed as gallic acid equivalent (GAE).

#### 2.6.2. Total Flavonoid Content (TFC)

The NaNO_2_-Al(NO_3_)_3_ colorimetric method was used to determine the TFC of the samples according to the method of Fu et al. [20] with a slight modification. 1 mL of extract was mixed with 0.4 mL of 5% NaNO_2_ solution and kept for 6 min, then 0.4 mL of 10% Al(NO_3_)_3_ solution was added and kept for 6 min, and 4 mL of 4% NaOH solution was added and kept for 15 min after shaking. Then, 4.2 mL of 4% NaOH solution was added and adjusted to 10 mL. After vortexing, the absorbance was measured at 510 nm. The results were expressed as rutin equivalents (RE).

#### 2.6.3. HPLC-PDA Analysis

Phenolic compounds were determined by the method of Liu et al. [21]. A total of 10 mL of sample was taken to make a methanol extract by the method of 2.6.1 and passed through a 0.45 μm filter membrane. An HPLC system (1260 Infinity II, Agilent Technologies Ltd., Shanghai, China) equipped with a photodiode array tube (PDA) and a C18 (250 mm × 4.6 mm, Shanghai Huifen Scientific Instruments Co., Ltd., Shanghai, China) column was used. The column temperature was 30 °C, the mobile phase consisted of 0.1% phosphoric acid solution (phase A) and methanol (phase B), and the absorbance was measured at 330 nm. Gallic acid, protocatechuic acid, chlorogenic acid, p-coumaric acid, ferulic acid, catechin, epicatechin, kaempferol, quercetin, isorhamnetin, and Prunus amygdalus were used as the standards. The results were expressed in mg/L.

#### 2.6.4. DPPH Free Radical Scavenging Capacity

The free radical scavenging ability of sample DPPH was determined using the method of Liu et al. [22]. In the experimental group, 100 μL of sample extract was mixed with 100 μL of 0.1 mM DPPH solution and kept in the dark at ambient temperature for 30 min. The absorbance was measured at 517 nm. In the control group, 100 μL of sample methanol extract was mixed with 100 μL of methanol, and in the blank group, 100 μL of methanol was mixed with 100 μL of DPPH solution. The calculation formula was:

(6) $$Scavenging\;rate\left(\%\right)=\frac{1-\left(A_{1}-A_{2}\right)}{A_{0}}\times 100$$

where *A*_1_ is the experimental group, *A*_2_ is the control group, and *A*_0_ is the blank group.

#### 2.6.5. ABTS Free Radical Scavenging Capacity

ABTS free radical scavenging capacity was assayed according to the method of Thaipong et al. [23] with slight modifications. The ABTS working solution was obtained by mixing ABTS solution (4 mM) with K_2_S_2_O_8_ solution (6 mM) at a ratio of 1:1 (*v*/*v*). The mixture was kept in the dark for 12 h and then diluted with deionized water until the absorbance at 734 nm reached 0.7 ± 0.02. In the experimental group, 100 μL of the sample was mixed with 100 μL of ABTS working solution, and the absorbance at 734 nm was measured after being kept for 6 min. Methanol was used instead of the sample methanol extract for the blank group. ABTS radical scavenging capacity was calculated as follows:

(7) $$Scavenging\;rate\left(\%\right)=\frac{A_{C}-A_{S}}{A_{C}}\times 100$$

where *A_C_* is the blank group and *A_S_* is the experimental group.

#### 2.6.6. α-Glucosidase (α-Glu) Inhibition Rate

For the determination of the α-Glu inhibition rate, refer to the method of Su et al. [24]; 100 μL PBS (0.1 mol/L, pH 6.8) and 100 μL of α-Glu solution (1 U/mL in 0.1 mol/LPBS) were mixed with 100 μL of the sample and then preheated at 37 °C for 10 min. Then, 100 μL of pNPG was added and reacted at 37 °C for 20 min, and then 500 μL of NaCO_3_ (1 mol/L) was added to terminate the reaction, and the absorbance at 405 nm was measured. The inhibition rate was calculated as follows:

(8) $$Inhibition\;activity\left(\%\right)=\frac{1-\left(A_{sample}-A_{sample\;background}\right)}{A_{control}-A_{blank}}\times 100$$

where *A_sample_* is the sample group, *A_sample background_* is the sample blank group, *A_control_* is the control group, and *A_blank_* is the blank group.

#### 2.6.7. α-Amylase (α-Amy) Inhibition Rate

The inhibition rate of α-Amy was determined by referring to the method of Su et al. [24], 50 μL of sample and 50 μL of α-Amy (2 U/mL) were mixed and preheated at 37 °C for 10 min. Then, 50 μL of soluble starch solution (1 mg/mL) was added to react at 37 °C for 20 min, and then the reaction was terminated by the addition of 50 μL of DNS. The inhibition rate was calculated according to Equation (8).

### 2.7. Sensory Evaluation of S-FCP

A total of 3 g S-FCP was mixed with 100 mL water (60 ± 5 °C) and provided to the sensory evaluation team after the temperature cooled to about 40 °C. The sensory evaluation team consisted of 18 trained food professionals (11 women, 7 men, 22–35 years old). Then, the nine-point hedonic evaluation system was used to evaluate the color, odor, taste, and overall acceptability of the rehydrated sample, ranging from very dislike = 1 to very like = 9 [25].

### 2.8. Statistical Analysis

Each group of samples was carried out in triplicate, and the results were exhibited as the mean ± standard deviation. The quantitative data were analyzed by one-way analysis of variance (ANOVA) with Tukey’s test using SPSS 29.0 (IBM, Armonk, NY, USA), and differences were considered significant when *p* < 0.05. Plots were performed with Origin 2019 (OriginLab Corp., Northampton, MA, USA).

## 3. Results and Discussion

### 3.1. Physicochemical Properties of S-FCP

#### 3.1.1. Color Parameters

**Figure 1:**
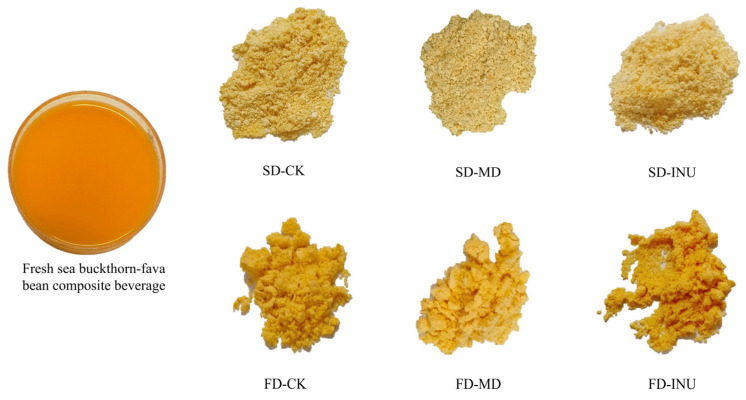
Fresh sea buckthorn–fava bean composite beverage, spray-dried and freeze-dried S-FCP with different carriers added.

Color parameters are important indicators of food acceptability. Figure 1 shows the appearance of fresh sea buckthorn–fava bean composite beverage and spray-dried and freeze-dried S-FCP with the addition of different carriers, and the corresponding color indexes are listed in Table 1. It can be observed that the L* value of the fresh sea buckthorn–fava bean composite beverage was 53.99 ± 0.09. By comparison, the L* values of the S-FCP increased significantly (*p* < 0.05), indicating that both spray-drying and freeze-drying could result in higher brightness. In both dried powder samples, the L* values of the FD samples (71.71 ± 0.11 to 74.39 ± 0.23) were lower than those of the SD samples (81.11 ± 0.01 to 83.72 ± 0.04), which was in line with the findings of Dincer and Temiz [6], suggesting that the color of the powder samples is related to the particle structure and the space left by ice crystal sublimation leading to the darker surface of the FD particles. It was also observed that samples containing MD had the highest L* values in both the SD and FD treatment groups, indicating that the addition of different carriers also affected the color of the samples [26].

The a* and b* values of the samples in this study were positive, indicating the reddish and yellowish coloration of the samples. Compared to the a* values of fresh sea buckthorn–fava bean composite beverage (33.62 ± 0.14), the a* values of FD samples (ranging from 29.14 ± 0.13 to 32.72 ± 0.11) showed relatively small changes, while the a* values of SD samples (ranging from 15.86 ± 0.02 to 18.12 ± 0.02) were significantly lower (*p* < 0.05), indicating that the red coloration of SD samples was significantly weakened, which might be the result of the high temperature of spray drying destroying the heat-sensitive natural colored substances, such as carotenoids and anthocyanins, in the S-FCP. Similar results were observed in spray-dried fire-ratchet powder, composite berry powder, and pomegranate powder [6,27,28], whereas the low-temperature, low-oxygen, and micro-pressure environments of freeze-drying could better preserve the colored substances in the samples. For the b* values, the FD samples (72.51 ± 0.65 to 78.67 ± 0.39) were significantly higher than those of the fresh sea buckthorn–fava bean composite beverage (62.58 ± 0.24), indicating a deepening yellow color of the FD samples, which is in line with the natural attributes of the product, resulting in the product possessing a more desirable appearance. In addition, among the two drying treatment groups, the a* and b* values of the samples containing INU were relatively larger compared to the samples containing MD, and thus the samples containing INU had a darker red and yellow color, suggesting that the INU group was able to better maintain the color of the product compared to the MD group.

The total color difference in the S-FCP compared to the fresh sea buckthorn–fava bean composite beverage (∆*E* values) shows that the FD samples (23.00 ± 0.08 to 24.41 ± 0.25) were significantly smaller (*p* < 0.05) than the SD samples (31.37 ± 0.12 to 34.80 ± 0.14), suggesting that the color of the FD samples was closer to the color of the fresh sea buckthorn–fava bean composite beverage. The change in the ∆*E* value of SD samples in this study was mainly due to the increase in L* value and the decrease in a* value (increase in brightness and decrease in red color), while the change in the ∆*E* value of FD samples was mainly due to the increase in L* value and the increase in b* value (increase in brightness and deepening of the yellow color). Therefore, freeze-drying technology and using INU as the carrier are favorable in achieving the desired color of S-FCP.

#### 3.1.2. Particle Size

**Figure 2:**
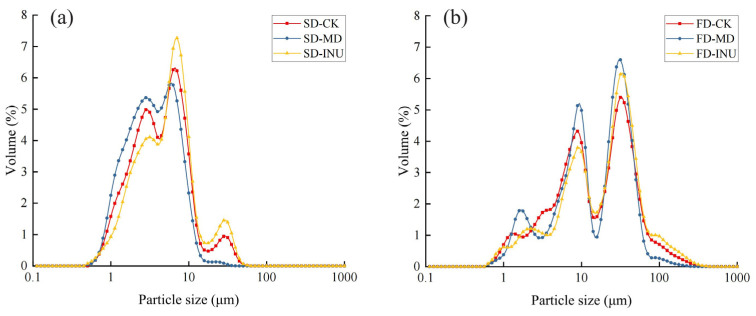
(**a**) Particle size distribution of spray-dried S-FCP with different carriers added; (**b**) Particle size distribution of freeze-dried S-FCP with different carriers added.

Particle size is an important parameter to measure the size of particulate matter, and particle size is closely related to the rehydration characteristics of instant powder [29]. Figure 2 shows the particle size distribution of spray-dried and freeze-dried S-FCP with different carriers added. All the samples showed multiple peaks, among which the FD samples were more dispersed compared to the SD samples. As can be seen in Table 2, the span values of the FD samples (2.80 ± 0.01 to 3.31 ± 0.01) were larger than those of the SD samples (2.03 ± 0.07 to 2.17 ± 0.02), and the span values of the two samples were all >1. These results indicated that the FD samples exhibited a wider particle size distribution. This might be attributed to the fact that FD powders were obtained by mechanical milling and their particle size distribution depends mainly on the quality of that mechanical milling [30]. In addition, Table 2 also responds to the volumetric mean particle size D_[4,3]_ of the S-FCP, which can be seen with a range from 5.05 ± 0.09 μm to 6.86 ± 0.03 μm for SD samples, and 21.25 ± 0.10 μm to 24.61 ± 0.02 μm for FD samples, where SD samples presented smaller mean particle sizes. This was attributed to the higher spray pressure during the spray drying process, which resulted in an accelerated material ejection rate, leading to atomized droplets with smaller particle sizes; similar findings have been found in other studies [9,12,30]. The results of this study showed that the particle size of the instant powder was mainly affected by the drying method, while the effect of the carrier was relatively small.

#### 3.1.3. Microstructure

**Figure 3:**
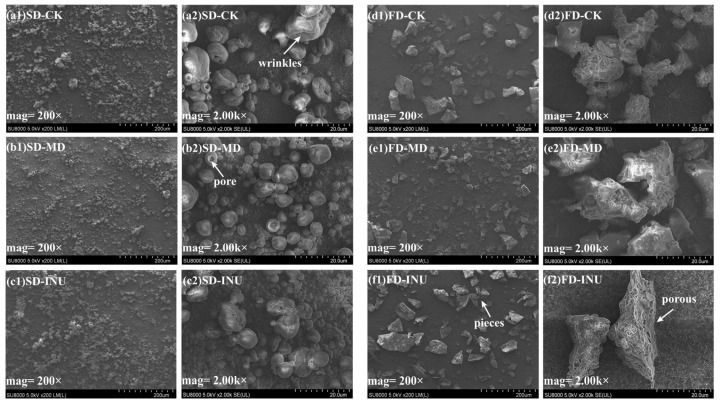
Scanning electron micrographs of spray-dried and freeze-dried S-FCP with the addition of different carriers.

The microstructure of powder particles is closely related to the rehydration characteristics of dried products [29]. Figure 3 shows the scanning electron microscope images of spray-dried and freeze-dried S-FCP with different carriers. Apparent differences in morphological characteristics could be observed from the SD and FD samples (Figure 3). The SD samples are mainly in the form of microspheres, and some of the particles have inwardly collapsing wrinkles on the surface, which is caused by the evaporation of water from the particles during the spray drying process, and the faster the rate of evaporation of water is, the smoother the surface of the particles is [31]. In addition, most of the SD sample particles showed pores on the surface, which further confirmed that the SD sample particles were hollow spherical structures. The FD sample particles were mainly porous or shaped like pieces of broken glass, which could be attributed to the formation of ice crystals during the pre-frozen stage, and the sublimation of ice crystals would leave numerous pores, which were conducive to increasing the surface area of the particles, and ultimately forming a loose and porous cake-like microstructure, and then the lyophilized material would be converted into particles with different shapes and sizes by mechanical grinding, which was a typical morphological characteristic of FD sample particles, as previously reported [32,33,34]. For the SD treatment group, differences could be observed concerning the particle morphology of the S-FCP with different carriers. Figure 3(a1–c1) showed that SD-CK and SD-INU particles showed an obvious agglomeration phenomenon, while no similar phenomenon was found in SD-MD particles, which may be related to the nature of the carrier and the positive correlation of hygroscopicity of the powder as previously reported. A similar phenomenon was also observed in blueberry powder with differing carriers by Araujo-Díaz et al. [35]. They found that the higher the water activity of the powder, the more obvious the agglomeration phenomenon, but at the same water activity, the agglomeration phenomenon of the sample containing MD was weaker than that of the sample containing INU. Therefore, in addition to the drying method, the addition of different carriers will also affect the microscopic morphology of the powder.

#### 3.1.4. Contact Angle, Wetting Time, and Solubility

**Figure 4:**
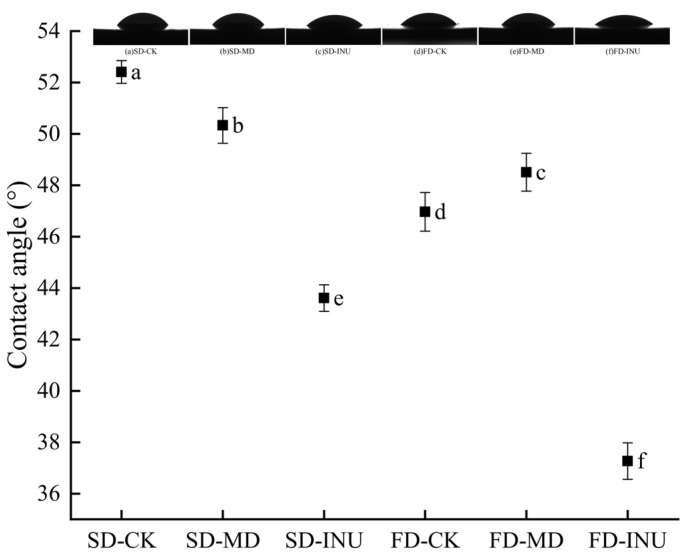
Contact angles of spray-dried and freeze-dried S-FCP with different carriers added. Different letters in the figur indicate significant differences in values (*p* < 0.05).

The contact angle is the angle of the tangent line between the sample surface and the droplet contact point, and a smaller contact angle represents a more wettable sample [36]. Wetting time is also an important indicator to indirectly reflect the wetting of the sample: the shorter the wetting time per unit mass of the sample, the stronger the wetting of the sample [29,37]. As can be seen from Table 3 and Figure 4, the contact angle and wetting time of the FD sample without added carrier (FD-CK) were 10.37% and 18.03% smaller than those of the SD sample without added carrier (SD-CK), respectively, indicating that the FD-CK was more wettable than the SD-CK. This might be because the FD sample has a larger particle size and a porous structure, which is more conducive to the infiltration of water molecules into the powder structure [38]. Therefore, FD products exhibited better wettability than SD ones. In addition, in the SD treatment group, the contact angle and wetting time of SD-INU were the smallest at 52.41 ± 0.15° and 81.34 ± 0.35 s. This might be due to higher hydrophilic group content derived from fructans units in INU, which led to an increasing force between the sample and the water so that the contact angle and wetting time of the sample were reduced [39]. Similarly, in the FD-treated group, the contact angle and wetting time of FD-INU were the smallest, measuring 37.27 ± 0.19° and 74.19 ± 0.54 s, respectively. However, the contact angle and wetting time of FD-MD increased compared to FD-CK, indicating that the wetting of FD samples with maltodextrin was reduced compared to FD samples without added carrier. This is similar to the results of the study of Zotarelli et al. [12], who found that, compared to mango powder without maltodextrin, the addition of maltodextrin as a carrier increased the wetting time. Thus, apart from the micromorphology and particle size of the powder, the surface composition of the powder also affects the sample’s wettability.

Solubility is an important index for evaluating the hydration characteristics of powder samples which is closely related to the practical application of S-FCP. The solubility of the powder samples was affected by the composition of the samples, the micro-morphology of the particles, the particle size, etc. The higher the solubility, the higher the quality of the product [6]. As shown in Table 3, the solubility of the S-FCP was mainly affected by the drying method, and it was found that the solubility of the FD samples (ranging from 52.24 ± 0.36% to 54.42 ± 0.51%) was significantly higher than that of the SD samples (ranging from 39.09 ± 0.44% to 41.54 ± 0.48%) (*p* < 0.05), which may be due to the high temperature causing the S-FCP to denature the broad bean protein molecules. Burger et al. [40] found a similar phenomenon in pea isolate proteins, where they found that high temperatures led to the denaturation and aggregation of pea isolate proteins, which led to an increase in their hydrophobicity. In addition, the solubility increased in both treatment groups after the addition of the carrier and was relatively better with the addition of INU, which may be related to the hydrophilicity of INU. Therefore, based on the results of contact angle, wetting time, and solubility, the freeze-drying technique coupled with the use of INU as a carrier was more suitable for the production of S-FCP.

#### 3.1.5. Moisture Content and Distribution

The moisture content of the powder samples directly affected the stability of the samples. Table 3 responds to the moisture content of spray-dried and freeze-dried S-FCP with different carriers added. The results showed that the moisture content of the SD samples (1.81 ± 0.05% to 3.09 ± 0.05%) was lower compared to that of the FD samples (3.57 ± 0.04% to 4.97 ± 0.03%). The reason for this difference may be that the high temperature of SD makes the internal thermal conductivity of the powder faster, which favors the evaporation of powder moisture [41]. There were also differences in moisture content for samples that were dried using the same drying method but with different carriers added. In both drying treatment groups, the moisture content of the samples containing MD was much lower than that of the samples containing INU (*p* < 0.05), which might be attributed to the change in the hygroscopicity of the powder with the addition of the carrier, and it has been found that INU is more hygroscopic compared to MD because it carries more hydrophilic groups and absorbs moisture from the air more easily [42]. Other studies have also found that maltodextrin-added powder samples have relatively lower moisture content compared to carriers such as inulin, gum arabic, corn starch, and pectin [43,44]. In addition, the moisture content of all the samples in this study was lower than 6%, which can minimize the phenomenon of agglomeration and caking of powder samples, which is conducive to the improvement of sample stability and the prolongation of sample shelf life [45].

**Figure 5:**
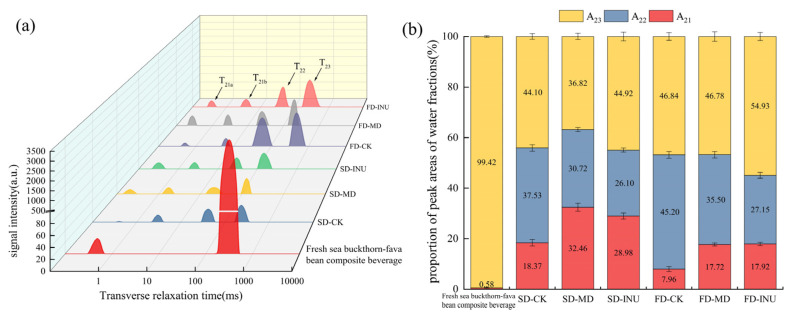
(**a**) Relaxation time (T_2_) inversion profiles of fresh sea buckthorn–fava bean composite beverage and spray-dried and freeze-dried S-FCP with the addition of different carriers (**b**) Relative peak areas of different water fractions in fresh sea buckthorn–fava bean composite beverage and spray-dried and freeze-dried S-FCP with the addition of different carriers.

LF-NMR is a technique that can detect the transverse relaxation time (T_2_) of hydrogen protons in samples, reflecting changes in moisture distribution. A shorter T_2_ indicates that the water molecules are more tightly bound to the substrate, which means that the degree of freedom of the hydrogen protons is lower, while a longer T_2_ stands for a higher degree of freedom of the hydrogen protons [46]. The signal amplitude corresponding to T_2_ is proportional to the relative content of hydrogen protons, i.e., the area of each peak of the T_2_ inversion pattern is proportional to the relative content of water in each state. As can be seen from Figure 5a, after the drying treatment, the peak number of the sample changed from 2 to 4, and each wave peak represented a different form of moisture present. T_21a_ (0–1 ms) and T_21b_ (1–10 ms) represent strongly bound water and weakly bound water that are tightly bound to the macromolecules, respectively; T_22_ (10–100 ms) represents water that does not flow easily and that is less affected by the binding force, and T_23_ (100–1000 ms) represents the most mobile, free water. The relative peak areas A_21_, A_22_, and A_23_ corresponding to these peaks also changed; as can be seen in Figure 5b, A_21_ and A_22_ increased significantly in SD and FD samples compared to fresh sea buckthorn–fava bean composite beverage: A_21_ changed from 0.58% to the ranges of 18.37–32.46% (SD) and 7.96–17.92% (FD); A_22_ changed from 0% to the ranges of 26.10–37.53% (SD) and 27.15–45.20% (FD); A_23_ significantly decreased from 99.42% to the ranges of 36.82–44.92% (SD) and 46.78–54.93% (FD). The significant increase in A_21_ and A_22_ may be because, with the continuous removal of free water, the increasing concentration of the sample substrate strengthens the binding effect between water molecules and substrate macromolecules, which leads to the deterioration of water mobility [47]. The apparent reduction in A_23_ is due to the extreme mobility of free water, which can be rapidly removed during the initial stages of drying [48,49,50]. It can also be seen from Figure 5b that the A_21_ (7.96–17.92%) of the FD samples was relatively smaller compared with that of the SD samples (18.37–32.46%), which might be due to the formation of ice crystals when the sample was pre-frozen at −80 °C. These ice crystals sublimed during vacuum freeze-drying, leaving a large number of pores in the sample. As a result, bound water cannot bind closely to substrate macromolecules [51]. In addition, it was found that T_2_ in both drying treatment groups decreased and the wave peak shifted to the left side of the coordinate axis after the addition of MD and INU compared to SD-CK and FD-CK, indicating that the binding between the water molecules and the substrate macromolecules was enhanced, which was favorable for the storage stability of the samples.

#### 3.1.6. Characterization of Functional Groups

**Figure 6:**
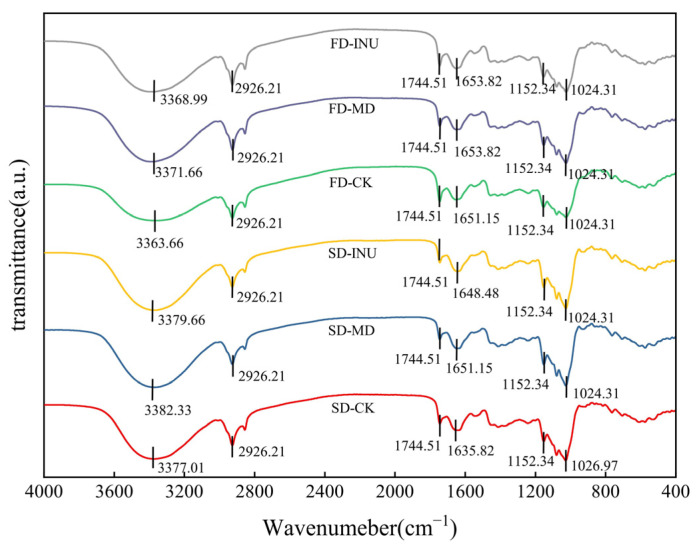
FT-IR spectra of spray-dried and freeze-dried S-FCP with the addition of different carriers.

The FT-IR technique reveals the molecular structure and chemical bonding characteristics of the different components and therefore can be used to analyze the molecular interactions between the carrier agent and the core material [52]. Figure 6 shows the FT-IR spectra of spray-dried and freeze-dried S-FCP with different carriers within the bands of 4000–400 cm^−1^. The FT-IR spectra obtained in this study have similar peaks characterized by functional groups as appeared in previous studies [9,26,41]. Significant absorption peaks were observed in the vicinity of 3363.66–3382.33 cm^−1^ for all samples, which could be attributed to the O-H stretching vibration. This was related to the hydrogen bond formed between the carrier and the composite powder, and the residual water in the composite powder [41]. Compared to the SD sample and the FD sample with added MD, the peak intensities of FD-CK and FD-INU weakened around 3363.66–3382.33 cm^−1^ and shifted to low wave numbers, indicating that the hydrogen bonding structures formed in the SD sample and the FD sample with added MD were stronger than the hydrogen bonding in FD-CK and FD-INU. The absorption peak at 2926.21 cm^−1^ is attributed to the C-H stretching vibration of olefins. This might be attributed to the high oil and fat content of sea buckthorn, comprising a significant proportion of unsaturated fatty acids, leading to the characteristic peak [9]. The characteristic peaks at 1744.51 cm^−1^, 1648.48–1653.82 cm^−1^, 1152.34 cm^−1^, and 1024.31–1026.97 cm^−1^ are due to the asymmetric contraction of -C=O in the carboxylate group, O-H bending of the C-O-H bond, C-O stretching, and folding vibration of C-O-C, respectively. These characteristic peaks at corresponding wave numbers possessing similar amplitudes indicated that the functional groups did not undergo significant changes. In addition, no additional characteristic peaks appeared in the samples with the addition of carriers compared with those without carriers (SD-CK and FD-CK), indicating that the carriers did not react chemically with the composition of S-FCP to form new compounds.

#### 3.1.7. Correlation Analysis

**Figure 7:**
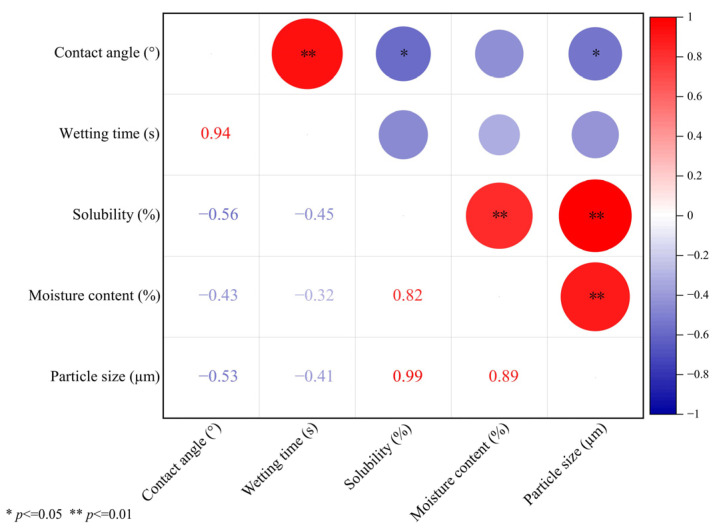
Correlation analysis between contact angle, wetting time, solubility, moisture content, and particle size of spray-dried and freeze-dried S-FCP with different carrier additions.

To determine the relationship between the contact angle, wetting time, solubility, moisture content, and particle size of spray-dried and freeze-dried S-FCP with different carriers added, Pearson correlation analysis was carried out, as shown in Figure 7. The contact angle was positively correlated with wetting time, but negatively correlated with solubility, moisture content, and particle size. Wetting time was negatively correlated with solubility, moisture content, and particle size. It was found that, when the powder samples have larger particles, the inter-particle mobility is enhanced, and water molecules are more likely to diffuse to the surface of the particles, which in turn enhances the wettability of the powder samples [29]. Solubility was significantly and positively correlated with moisture content and particle size, and the moisture content of powder samples significantly affected their solubility, with similar results reported by Aryaee et al. [28]. In addition, moisture content was significantly and positively correlated with particle size. It is worth noting that the conclusions of correlation analysis are not deterministic, but directional. This provides a basis for follow-up studies.

### 3.2. Bioactive Components, Antioxidant Capacity, and Hypoglycaemic Effect of S-FCP

#### 3.2.1. TPC and TFC

**Figure 8:**
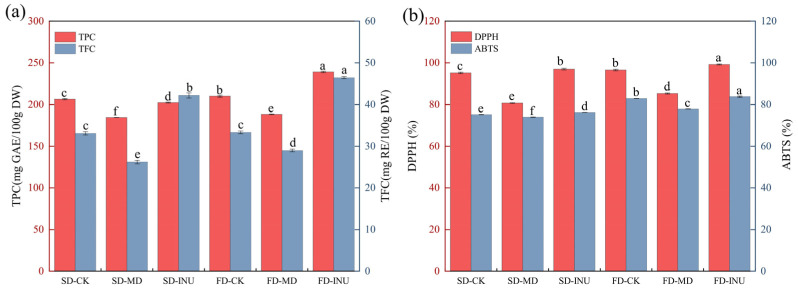
(**a**) TPC and TFC of spray-dried and freeze-dried S-FCP with different carriers added; (**b**) DPPH and ABTS radical scavenging capacity of spray-dried and freeze-dried S-FCP with different carriers. Different letters in different graph indicate significant differences in values (*p* < 0.05).

The total phenol content (TPC) and total flavonoid content (TFC) of spray-dried and freeze-dried S-FCP with different carriers are shown in Figure 8a. It can be seen that different carriers and drying methods have obvious effects on the TPC and TFC of samples, among which the TPC and TFC of FD-INU are the highest, which are 239.15 ± 0.82 mgGAE/100 g and 46.44 ± 0.26 mg RE/100 g, respectively. SD-MD had the lowest TPC and TFC (184.27 ± 0.12 mgGAE/100 g and 26.21 ± 0.42 mg RE/100 g, respectively). It was also observed that the TPC of FD-CK was higher than that of SD-CK, possibly because the ice crystals formed in the freeze-drying process caused the rupture of plant cells and promoted the release of phenolic substances, and the high temperature of spray drying also promoted the degradation of phenolic substances [28]. Compared to the sample without carrier, the TPC and TFC of the sample after adding MD were significantly decreased (*p* < 0.05), which might be due to the concentration effect of the carrier, that is, as the percentage of MD in the instant powder per unit mass increased, the percentage of the raw material base decreased, resulting in a decrease in TPC and TFC [11]. The higher TPC and TFC of the samples with INU addition can be attributed to the fact that inulin has a high glass transition temperature and a flexible skeletal structure, making it an effective stabilizer of bioactive ingredients. In contrast, the stabilizing effect of maltodextrins on phenolics and flavonoids is mainly dependent on their glucose equivalents (DE), with a higher DE resulting in lower retention of the compounds. This is under the findings of Tkacz et al. [8]. Therefore, FD-INU has the highest total phenolic and total flavonoid content.

#### 3.2.2. Phenolic Compound

Phenolic compounds are secondary metabolites of plants and are of value due to their beneficial effects on human health [53]. Given that FD-INU had the highest total phenolic and total flavonoid contents, the determination of phenolic compounds was conducted in sea buckthorn juice, fresh sea buckthorn–fava bean composite juice, and FD-INU rehydrated samples, as illustrated in Table 4. A total of 11 phenolic compounds were identified. The chromatograms of the samples are shown in Supplementary Figures S1–S3. The findings indicated that the concentration of each phenolic compound was diminished in the fresh sea buckthorn–fava bean composite juice relative to the sea buckthorn juice. However, the decline in catechins and epicatechins was not statistically significant (*p* > 0.05). This may be due to the formation of flavan-3-ols from condensed tannins in the fava beans by depolymerization during hot pulping, and the release of bound catechins from heating, which has been reported in a previous study [54]. Furthermore, it was observed that, except for protocatechuic acid, the contents of all other phenolic compounds in the rehydrated samples of FD-INU exhibited a slight decrease compared to the fresh sea buckthorn–fava bean composite juice. However, among them, the content of protocatechuic acid (ranging from 5.29 ± 0.41 mg/L–5.67 ± 0.30 mg/L) increased slightly, probably due to the conversion of certain components of sea buckthorn to protocatechuic acid, such as ethyl protocatechuate, during freeze-drying [55]. In conclusion, FD-INU did not result in a significant loss of phenolic compounds.

#### 3.2.3. DPPH and ABTS Scavenging Capacity

Figure 8b shows the results of the antioxidant capacity of S-FCP as determined by the DPPH and ABTS scavenging method. The DPPH and ABTS scavenging capacities of FD-CK were 1.47% and 9.37% higher than those of SD-CK, respectively, and the order of the DPPH and ABTS scavenging capacities in both drying treatment groups was INU > CK > MD. This is consistent with the TPC and TFC results, probably because phenolics and flavonoids have hydrogen donating or electron transferring abilities, so the radical scavenging ability of the samples was related to the retention of phenolics and flavonoids [11,48]. Therefore, FD-INU has a stronger antioxidant capacity.

#### 3.2.4. In Vitro Hypoglycemic Effect

**Figure 9:**
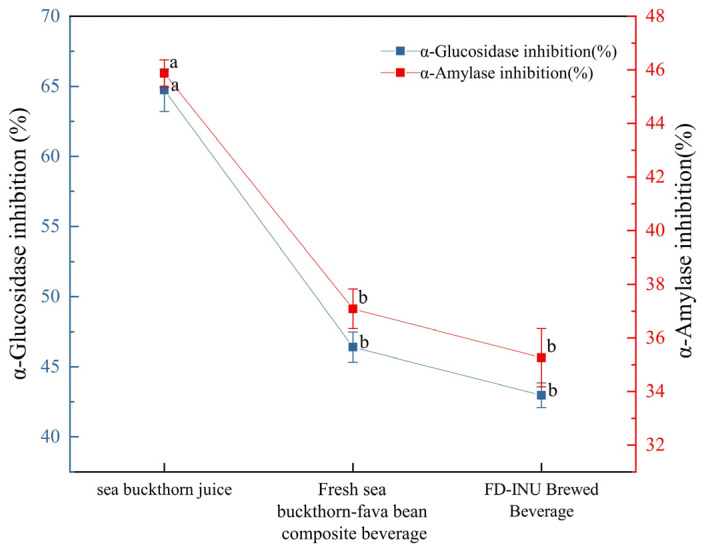
Inhibition of α-Glu and α-Amy by sea buckthorn juice, fresh sea buckthorn–fava bean composite beverage, and FD-INU rehydration beverage. Different letters in the figur indicate significant differences in values (*p* < 0.05).

α-Glu and α-Amy are essential carbohydrate-hydrolyzing enzymes and therefore key targets in the regulation of postprandial glucose, and polyphenols are good enzyme inhibitors [24]. In this study, the inhibition rates of α-Glu and α-Amy were determined in polyphenol-rich sea buckthorn juice, fresh sea buckthorn–fava bean composite juice and FD-INU rehydrated samples, as shown in Figure 9. It can be observed that sea buckthorn juice showed the highest inhibition of both enzymes. Compared to fresh sea buckthorn–fava bean composite juice, the inhibition rate of FD-INU rehydrated samples on both enzymes decreased, but the difference was not significant (*p* > 0.05). This result might be related to the content and composition of phenolic compounds, which have been shown to have potential dual inhibitory effects on α-Glu and α-Amy [56]. Compared to the other supplements, FD-INU rehydrated samples showed higher inhibition of α-Glu than probiotic yogurt supplemented with mango peel powder [57], probiotic isolates from papaya fermentation broth [58], and others. Thus, the use of FD-INU as a dietary supplement in the daily diet may improve postprandial blood glucose levels to some extent.

### 3.3. Sensory Evaluation

**Figure 10:**
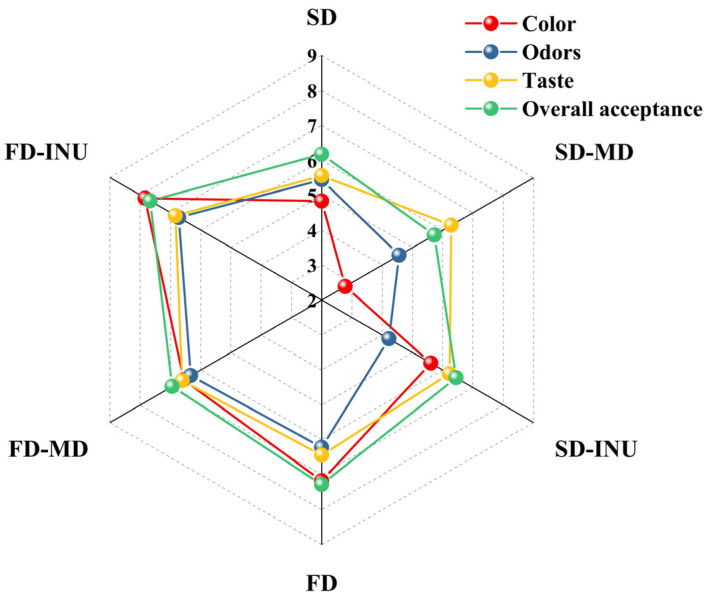
Sensory evaluation of spray-dried and freeze-dried S-FCP rehydrated with different carriers.

The sensory evaluation of the sample enables an understanding of the product’s acceptability to the consumer. As illustrated in Figure 10, the FD sample exhibited higher scores for the color and odor attributes following rehydration in comparison to the SD sample. FD-INU exhibited the highest color score (7.83) and odor score (6.72). About the taste attribute scores, no significant difference was observed following the rehydration of all samples, except SD-CK (*p* > 0.05). However, samples with carrier added exhibited higher scores in comparison to those without carrier. Concerning overall acceptability, FD-INU exhibited the highest score (7.67) following rehydration. The results of the sensory evaluation indicated that the low temperature and low oxygen environment of freeze-drying were more conducive to changes in the product’s color, aroma, and taste. Furthermore, the addition of a carrier was conducive to an improvement in the product’s taste, thereby enhancing its overall acceptability.

## 4. Conclusions

In summary, the freeze-drying technique and the use of INU as a carrier were the best solutions for the production of S-FCP in terms of color, solubility, nutritional quality, and sensory evaluation, but there were still shortcomings. Therefore, the quality of instant powder can be improved in the future by using new drying techniques such as spray freeze-drying or replacing a single carrier with a composite carrier. Meanwhile, further investigation on the stability of the product during storage and the bioavailability of the nutrients during in vitro or in vivo digestion of the product would be valuable for the study of instant powder.

## Figures and Tables

**Table 1:** Color parameters of fresh sea buckthorn–fava bean composite beverage, spray-dried and freeze-dried S-FCP with different carriers added.

Samples	Color Parameters			
	L*	a*	b*	∆*E*
Fresh sea buckthorn–fava bean composite beverage	53.99 ± 0.09 ^g^	33.62 ± 0.14 ^a^	62.58 ± 0.24 ^e^	0.00 ± 0.00 ^f^
SD-CK	81.11 ± 0.01 ^c^	18.12 ± 0.02 ^e^	65.51 ± 0.03 ^d^	31.37 ± 0.12 ^c^
SD-MD	83.72 ± 0.04 ^a^	15.86 ± 0.02 ^g^	59.19 ± 0.03 ^f^	34.80 ± 0.14 ^a^
SD-INU	82.92 ± 0.01 ^b^	16.48 ± 0.03 ^f^	65.06 ± 0.03 ^d^	33.72 ± 0.15 ^b^
FD-CK	71.71 ± 0.11 ^f^	29.14 ± 0.13 ^d^	77.01 ± 0.18 ^b^	23.30 ± 0.08 ^e^
FD-MD	74.39 ± 0.23 ^d^	29.93 ± 0.31 ^c^	72.51 ± 0.65 ^c^	23.00 ± 0.08 ^e^
FD-INU	72.32 ± 0.03 ^e^	32.72 ± 0.11 ^b^	78.67 ± 0.39 ^a^	24.41 ± 0.25 ^d^

Note: Data are expressed as mean ± standard error (n = 3); different letters in the same column indicate significant differences in values (*p* < 0.05). SD-CK-spray-drying without added carrier; SD-MD-spray-drying with maltodextrin; SD-INU-spray-drying with inulin; FD-CK-freeze-drying without added carrier; FD-MD-freeze-drying with maltodextrin; FD-INU-freeze-drying with inulin.

**Table 2:** Particle size of spray-dried and freeze-dried S-FCP with different carriers added.

Samples	Particle Size	
	D_[4,3]_ (μm)	Span
SD-CK	5.71 ± 0.04 ^e^	2.17 ± 0.02 ^d^
SD-MD	5.05 ± 0.09 ^f^	2.03 ± 0.07 ^d^
SD-INU	6.86 ± 0.03 ^d^	2.09 ± 0.10 ^d^
FD-CK	23.95 ± 0.07 ^b^	3.31 ± 0.01 ^a^
FD-MD	21.25 ± 0.10 ^c^	2.80 ± 0.01 ^c^
FD-INU	24.61 ± 0.02 ^a^	3.02 ± 0.02 ^b^

Different letters in the same column indicate significant differences in values (*p* < 0.05).

**Table 3:** Moisture content, contact angle, wetting time, and solubility of spray-dried and freeze-dried S-FCP with the addition of different carriers.

Samples	Contact Angle (°)	Wetting Time (s)	Solubility (%)	Moisture Content (%)
SD-CK	52.41 ± 0.15 ^a^	115.98 ± 0.70 ^a^	39.09 ± 0.44 ^d^	3.09 ± 0.05 ^d^
SD-MD	50.33 ± 0.12 ^b^	102.87 ± 0.56 ^b^	40.02 ± 0.27 ^d^	1.81 ± 0.05 ^f^
SD-INU	43.61 ± 0.11 ^e^	81.34 ± 0.35 ^d^	41.54 ± 0.48 ^c^	2.65 ± 0.04 ^e^
FD-CK	46.97 ± 0.75 ^d^	95.07 ± 0.71 ^c^	52.24 ± 0.36 ^b^	4.97 ± 0.03 ^a^
FD-MD	48.51 ± 0.16 ^c^	103.37 ± 0.66 ^b^	52.96 ± 0.12 ^b^	3.57 ± 0.04 ^c^
FD-INU	37.27 ± 0.19 ^f^	74.19 ± 0.54 ^e^	54.42 ± 0.51 ^a^	4.41 ± 0.04 ^b^

Different letters in the same column indicate significant differences in values (*p* < 0.05).

**Table 4:** Phenolic compounds in sea buckthorn juice, fresh sea buckthorn–fava bean composite beverage, and FD-INU rehydration beverage (mg/L).

Phenolic Compound	Sample		
	Sea Buckthorn Juice	Fresh Sea Buckthorn–Fava Bean Composite Beverage	FD-INU Brewed Beverage
Gallic acid	39.72 ± 1.05 ^a^	21.43 ± 0.74 ^b^	18.25 ± 0.45 ^c^
Protocatechuic acid	8.23 ± 0.40 ^a^	5.29 ± 0.41 ^b^	5.67 ± 0.30 ^b^
Chlorogenic acid	12.59 ± 0.72 ^a^	7.58 ± 0.33 ^b^	6.42 ± 0.35 ^b^
p-Coumaric acid	13.24 ± 0.61 ^a^	8.46 ± 0.36 ^b^	7.03 ± 0.30 ^b^
Ferulic acid	3.65 ± 0.42 ^a^	2.51 ± 0.20 ^b^	1.89 ± 0.07 ^b^
Catechin	7.22 ± 0.41 ^a^	7.03 ± 0.28 ^a^	2.81 ± 0.40 ^b^
Epicatechin	5.46 ± 0.38 ^a^	5.25 ± 0.54 ^a^	1.74 ± 0.37 ^b^
Kaempferol	2.87 ± 0.22 ^a^	1.23 ± 0.14 ^b^	0.92 ± 0.09 ^b^
Quercetin	15.13 ± 0.44 ^a^	4.73 ± 0.21 ^b^	4.25 ± 0.34 ^b^
Isorhamnetin	18.25 ± 0.46 ^a^	5.65 ± 0.36 ^b^	4.87 ± 0.07 ^b^
Prunus amygdalus	4.37 ± 0.31 ^a^	1.42 ± 0.29 ^b^	1.25 ± 0.23 ^b^

Different letters in the same column indicate significant differences in values (*p* < 0.05).

## Data Availability

The original contributions presented in this study are included in the article/Supplementary Materials. Further inquiries can be directed to the corresponding authors.

## Supplementary Materials

The following supporting information can be downloaded at: https://www.mdpi.com/article/10.3390/foods13233944/s1. Figure S1: HPLC chromatogram of sea buckthorn juice; Figure S2: HPLC chromatogram of Fresh sea buckthorn–fava bean composite beverage; Figure S3: HPLC chromatogram of FD-INU rehydration sample.
