# Supplementary material for: Characterization of Physicochemical Properties, Bioactivities, and Sensory Attributes of Sea Buckthorn–Fava Bean Composite Instant Powder: Spray-Drying Versus Freeze-Drying Coupled with Carriers
Source: Foods. 2024 Dec 6;13(23):3944. doi: 10.3390/foods13233944 (PMC11641498; doi:10.3390/foods13233944)
Supplement: Supplementary file 1 [file foods-13-03944-s001.zip › foods-3351723-supplementary.pdf]

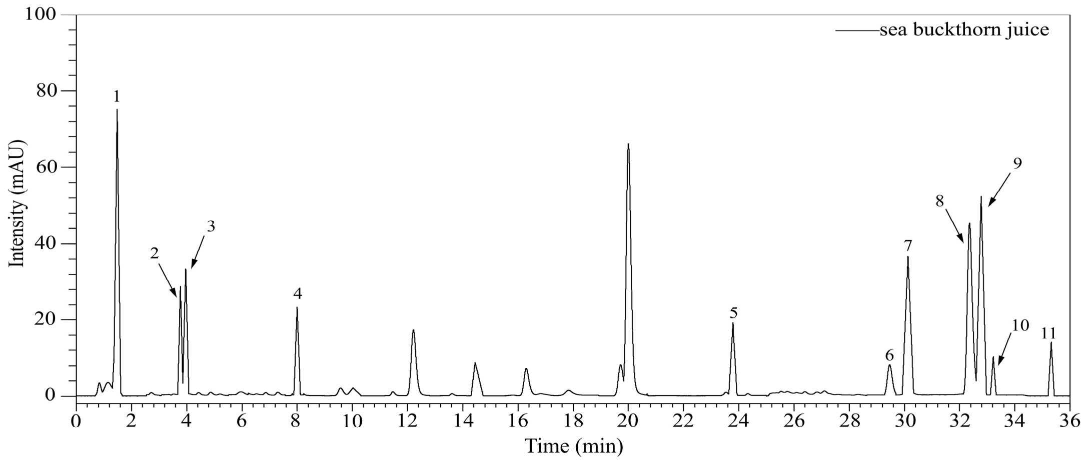

Figure S1. HPLC chromatogram of sea buckthorn juice. 1-Gallic acid; 2-Protocatechuic acid; 3-Chlorogenic acid; 4-Catechin; 5-Epicatechin; 6-Kaempferol; 7-p-coumaric acid; 8-Quercetin; 9-Isorhamnetin; 10-Ferulic acid; 11-Prunus amygdalus

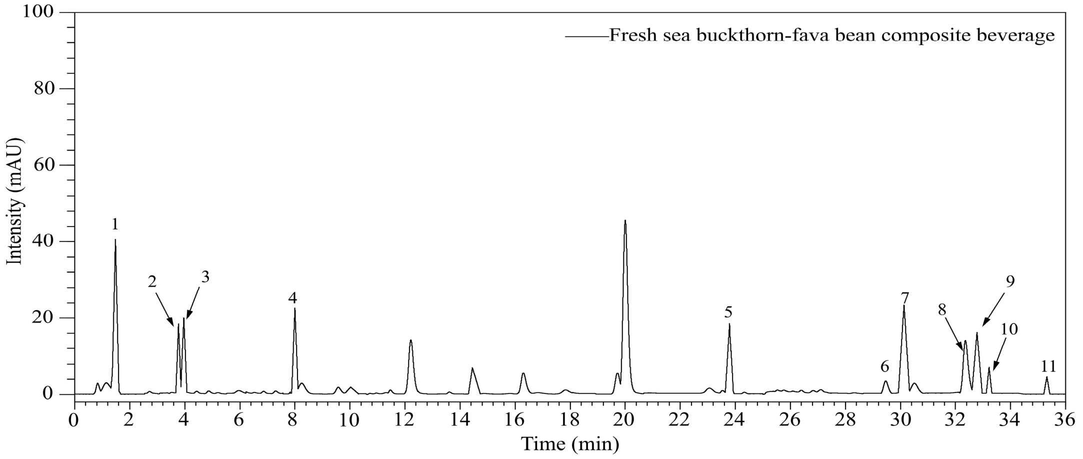

Figure S2. HPLC chromatogram of Fresh sea buckthorn-fava bean composite beverage. 1-Gallic acid; 2-Protocatechuic acid; 3-Chlorogenic acid; 4-Catechin; 5-Epicatechin; 6-Kaempferol; 7-p-coumaric acid; 8-Quercetin; 9-Isorhamnetin; 10-Ferulic acid; 11-Prunus amygdalus

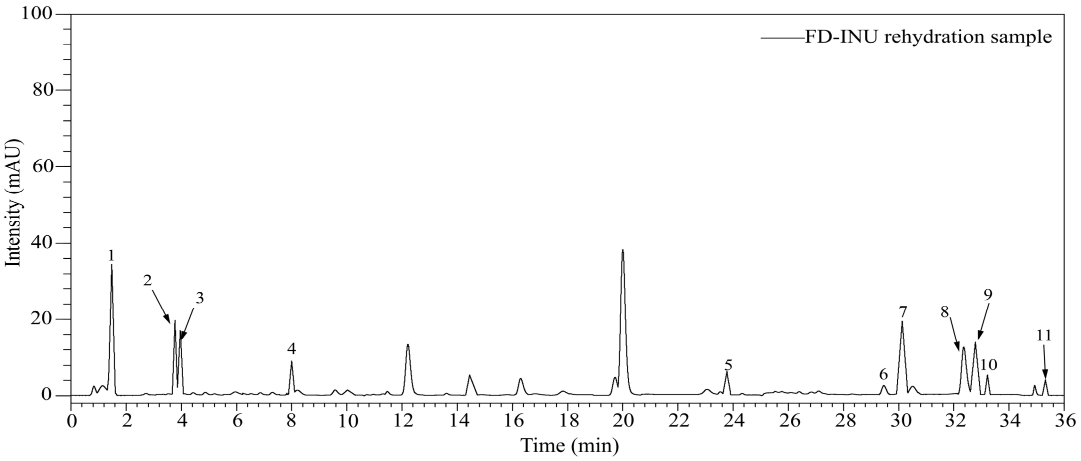

Figure S3. HPLC chromatogram of FD-INU rehydration sample. 1-Gallic acid; 2-Protocatechuic acid; 3-Chlorogenic acid; 4-Catechin; 5-Epicatechin; 6-Kaempferol; 7-p-coumaric acid; 8-Quercetin; 9-Isorhamnetin; 10-Ferulic acid; 11-Prunus amygdalus
